# Denitrification and Anammox in Tropical Aquaculture Settlement Ponds: An Isotope Tracer Approach for Evaluating N_2_ Production

**DOI:** 10.1371/journal.pone.0042810

**Published:** 2012-09-04

**Authors:** Sarah A. Castine, Dirk V. Erler, Lindsay A. Trott, Nicholas A. Paul, Rocky de Nys, Bradley D. Eyre

**Affiliations:** 1 AIMS@JCU, School of Marine and Tropical Biology, Australian Institute of Marine Science, Centre for Sustainable Tropical Fisheries and Aquaculture, James Cook University, Townsville, Queensland, Australia; 2 School of Environmental Science and Management, Centre for Coastal Biogeochemistry, Southern Cross University, Lismore, New South Wales, Australia; 3 Australian Institute of Marine Science, Townsville, Queensland, Australia; 4 School of Marine, Tropical Biology and Centre for Sustainable Tropical Fisheries and Aquaculture, James Cook University, Townsville, Queensland, Australia; Odum School of Ecology, University of Georgia, United States of America

## Abstract

Settlement ponds are used to treat aquaculture discharge water by removing nutrients through physical (settling) and biological (microbial transformation) processes. Nutrient removal through settling has been quantified, however, the occurrence of, and potential for microbial nitrogen (N) removal is largely unknown in these systems. Therefore, isotope tracer techniques were used to measure potential rates of denitrification and anaerobic ammonium oxidation (anammox) in the sediment of settlement ponds in tropical aquaculture systems. Dinitrogen gas (N_2_) was produced in all ponds, although potential rates were low (0–7.07 nmol N cm^−3^ h^−1^) relative to other aquatic systems. Denitrification was the main driver of N_2_ production, with anammox only detected in two of the four ponds. No correlations were detected between the measured sediment variables (total organic carbon, total nitrogen, iron, manganese, sulphur and phosphorous) and denitrification or anammox. Furthermore, denitrification was not carbon limited as the addition of particulate organic matter (paired *t*-Test; *P* = 0.350, *n* = 3) or methanol (paired *t*-Test; *P* = 0.744, *n* = 3) did not stimulate production of N_2_. A simple mass balance model showed that only 2.5% of added fixed N was removed in the studied settlement ponds through the denitrification and anammox processes. It is recommended that settlement ponds be used in conjunction with additional technologies (i.e. constructed wetlands or biological reactors) to enhance N_2_ production and N removal from aquaculture wastewater.

## Introduction

The release of anthropogenic N to the coastal zone poses a threat to many shallow marine ecosystems [1]. Discharge of aquaculture wastewaters has contributed to N enrichment of some coastal regions [2] and settlement ponds have been established as a remediation strategy from aquaculture wastewater prior to release to the environment [3], [4]. Settlement pond technologies are widely implemented as a low cost option for treating municipal [5], fish farm [6] and dairy farm wastewater [7]. However, the nutrient removal efficiency of settlement ponds associated with land-based tropical aquaculture systems is unclear. Generally, newly established (<1 yr old) settlement ponds, with a basic design, provide significant reductions in total suspended solids, but are less efficient in the remediation of dissolved nutrients [3], [8]. Furthermore, given that the efficiency of wetland wastewater treatment systems can decrease with age [9], it is likely that the performance of settlement ponds, which act as brackish water constructed wetlands, will decrease over time unless they are actively managed. Methods to improve the long term performance of tropical aquaculture settlement ponds include the use of extractive organisms such as algae, which can be cultured and subsequently harvested [10], and also the removal of settled organic rich particulates (sludge) which prevents remineralization of dissolved N back into the water column [3], [11]. Microbial nutrient transformation, which is largely un-quantified, also presents a potentially significant mechanism to reduce dissolved inorganic nitrogen (DIN) in aquaculture wastewater.

Denitrification and anammox are the major microbial processes removing fixed N from wastewater through the production of dinitrogen gas (N_2_). During denitrification, nitrate (NO_3_^−^) is reduced to nitrite (NO_2_^−^), nitric oxide (NO) and nitrous oxide (N_2_O), before eventually being converted to N_2_. Anammox also directly removes fixed N and couples NO_2_^−^ reduction with ammonium (NH_4_^+^) oxidation to produce N_2_ [12], [13]. Denitrification and anammox are also important for the removal of N from natural system such as intertidal flats [14], marsh sediments [15], deep anoxic waters [16] and sediments from the continental shelf (50 m) and slope (2000 m) [17]. Denitrification and anammox in natural systems can remove up to 266 mmol m^−2^ d^−1^ and 61 mmol m^−2^ d^−1^ of N, respectively [16]. These processes may be active in the treatment of aquaculture effluent water and could be exploited to enhance treatment. However, to date there has been no published quantification of denitrification and anammox in settlement pond systems treating waste from tropical aquaculture farms.

The first step in optimizing the removal of fixed N through the denitrification and anammox pathways is to quantify their activity in settlement ponds and relate this to the environment of the ponds. Accordingly, the aim of this study was to determine if denitrification and anammox occur in sediments collected from tropical settlement ponds that are used to treat effluent from commercial production of prawns (shrimp) and fish. We used sediment slurry assays to investigate potential N_2_ production in multiple zones of four settlement ponds on three farms (two prawn farms and one fish farm). We also investigated the relationship between the potential rates of N_2_ production with the geochemical characteristics of the ponds. Additionally, the effect of carbon on N_2_ production was tested since intensive aquaculture systems have N rich wastewaters where microbial N removal is typically limited by the supply of carbon as an electron donor [18]. Together these data provide new insight into N cycling processes in shallow tropical eutrophic marine systems in the context of N management.

## Methods 

### Study site

**Figure 1:**
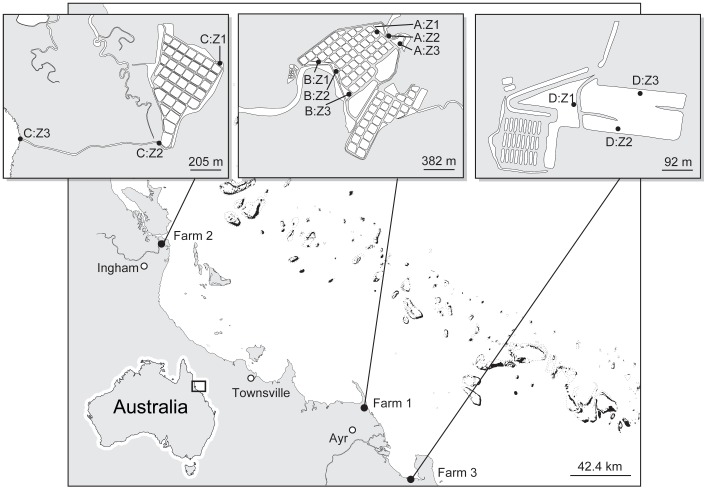
The location of three flow-through aquaculture farms along the North Australian coastline. The inset figures show the layout of each farm, the location of the settlement ponds and the 3 zones within each pond.

The presence of denitrification and anammox and their potential rates were measured in sediment collected from four settlement ponds across two operational prawn (*Penaeus monodon*) farms and one barramundi (*Lates calcarifer* Bloch) farm. At Farm 1 sediment was collected from the two functional settlement ponds, this allowed comparison of N_2_ production over small spatial scales (A and B; Figure 1). Additionally, sediment was collected from the only settlement pond at Farm 2 (Pond C) and the only settlement pond at Farm 3 (Pond D) (Figure 1). The three farms spanned the wet and dry tropics allowing comparison of N_2_ production in different environments. Each pond was split into 3 zones (Z1, Z2 and Z3) (Figure 1). In all ponds Z1 was near the inlet, Z2 was near the middle of the settlement pond, and Z3 was near the outlet of the settlement pond. Ponds have diurnal fluctuations in dissolved oxygen (DO) concentration; from <31.2 µM at night to supersaturation (>312.5 µM) during the day, indicating rapid water column productivity. Similarly, there are diurnal pH fluctuations (1–1.5 pH). According to farm records, salinity fluctuates seasonally, with dramatic decreases from 35‰ to 5‰ caused by heavy precipitation over the summer wet season. During the wet season access to the farms by road is limited. All assays were, therefore run within the same dry season, although salinity at Farm 2 was still reasonably low due to particularly heavy rainfall over the 2009/2010 wet season (see results section).

### Geochemical characteristics

To investigate the spatial variation of sediment characteristics within and between settlement ponds, and their role in driving N_2_ production, sediments were collected at Z1, Z2 and Z3 in each of the four settlement ponds (total of 12 zones) (Figure 1). Sampling was conducted in March 2010 for Ponds B and C and August 2010 for Ponds A and D. Directly before taking sediment samples, surface water salinity, temperature and pH were also measured at each zone within each pond using specific probes (YSI-Instruments). Probes were calibrated 24 h before use. They were submerged directly below the surface and left to stabilize for 5 min before recording data. A known volume of sediment (30–60 mL) was subsequently collected in intact sediment cores (*n* = 3 per zone). The sediments were extruded, weighed and subsequently oven dried (60°C) and reweighed for porosity (ø) determination (*n* = 3). Dried sediment was then milled (Rocklabs Ring Mill) for total N determination (LECO Truspec CN Analyzer). TOC was determined on a Shimadzu TOC-V Analyzer with a SSM-5000A Solid Sample Module. Solid phase S, P, Fe and Mn were also analyzed from milled sediment samples subjected to strong acid digestion. A THERMO Iris INTREPID II XSP ICP_AES was used to determine element content in triplicate sediment samples from each zone [19].

### Denitrification and anammox potential

Slurry assays were conducted to test for the presence of N_2_ (inclusive of both N_2_ and N_2_O) production through denitrification and anammox in March 2010 (Ponds B and C) or August 2010 (Ponds A and D). At the time of abiotic sample collection (see above), approximately 500 g of the most reactive sediments were collected from each zone in the four settlement ponds (*n* = 1 from each zone within each pond) (Figure 1) with a 30 mm i.d. corer [20]. The top 0–3 cm was collected because this includes the oxic and suboxic layers where NO_x_ is present or being reduced (denitrification) [21] and the anoxic layer below the interface, where NO_x_ penetrates but O_2_ does not, making conditions favorable for anammox [22]. Each sediment sample was placed into sterile plastic bags with minimal air and subsequently homogenized by hand and doubled bagged before transportation to the laboratory. Sediments remained in initial plastic bags at room temperature for up to five days until the start of the experiment. Standard anammox assays were run according to Trimmer et al. [23] and Thamdrup and Dalsgaard [22] with modifications (artificial seawater of the same salinity as site water) according to Erler et al. [20]. Artificial seawater was used to preclude the potential interference of ambient NO_3_^−^ in the isotope assay. A known volume of sediment (3–6 g) was loaded into Exetainers (Labco Ltd, High Wycombe, UK) and ∼5 mL of degassed (flushed for 1 hr with ultra pure He), artificial seawater was added to form a slurry. Sediments were pre-incubated (overnight) under anoxic conditions to ensure all residual NO_3_, NO_2_^−^ and O_2_ were consumed. Three different enrichment treatments (100 µM ^15^N-NH_4_^+^, 100 µM ^15^N-NH_4_^+^ plus 100 µM ^14^N-NO_3_^−^ or 100 µM ^15^N-NO_3_^−^) were added to the slurries. After the isotope amendment, the Exetainers were filled with the degassed seawater, capped without headspace and homogenized by inverting 2–3 times. Triplicate samples were sacrificed from each treatment at 0, 0.5, 17 h and 24 h by introducing 200 µL 50% w/v ZnCl_2_ through a rubber septum (*n* = 3). The 0 and 0.5 h time periods were chosen based on rapid turnover rates determined by Trimmer et al. [23] and 17 and 24 h were modified from Erler et al. [20]. Sacrifice of the slurry samples involved the addition of 2 mL He headspace to the samples through the septum. Samples were stored inverted and submerged in water at 4°C until analysis to ensure there was no diffusion of N_2_ into or out of the Exetainers. A gas chromatograph (Thermo Trace Ultra GC) interfaced to an isotope ratio mass spectrometer (IRMS, Thermo Delta V Plus IRMS) was used to determine ^29^N_2_ and ^30^N_2_ content of dissolved nitrogenous gas (includes ^15^N-N_2_ and ^15^N-N_2_O, collectively referred to as N_2_). Varying volumes (3–10 µL) of air were used as calibration standards.

The rate of N_2_ production in the 24 h incubation trials (above) was calculated from the slope of the regression over the incubation period (0, 0.5, 17, 24 h) based on Dalsgaard and Thamdrup [24]. However, in some cases the production of ^29^N_2_ and ^30^N_2_ was non-linear and rates were calculated based on the first two production points. Therefore a subsequent slurry assay was run to investigate N_2_ production rates over short, regular time intervals (15 min) to gain a more accurate insight into potential process rates. Sediment for the additional assays was collected from settlement Pond D, Zones 1 (*n* = 1) and 3 (*n* = 1) in October 2010. These zones were chosen because the production of N_2_ was non-linear during the 24 h incubation assay (see results section). Assays were run as described above, following the same sediment collection, pre-incubation, amendment and analysis techniques. However, samples were sacrificed at 0, 15, 30, 45 and 90 min.

### Slurry assay with carbon manipulation

The effect of an additional carbon source on the occurrence of denitrification and anammox was tested with a separate set of slurry assays because organic carbon limits N_2_ production in some aquaculture systems [25]. Extra sediment was collected in March and August (2010) in the sampling described above. Sediments from Ponds A (August) and C (March) were assayed with and without addition of a carbon source because organic carbon has stimulated or correlated with N_2_ production in some systems previously [17], [26], [27]. Concentrated particulate organic matter (POM) was used to test the effect of an *in situ* carbon source collected from Pond A. POM was collected by transporting settlement pond-influent water to the laboratory at the same time that sediments were collected. Suspended solids in influent water were concentrated by centrifugation (10 min at 3000 rpm). 400 µL aliquots of concentrated (∼100 mg L^−1^) POM were added to Exetainer vials prior to the addition of amendments. However, in the absence of a high total suspended solid load at Pond C, methanol (MeOH) was used as the carbon source as it stimulates denitrification but inhibits anammox in some circumstances [28], [29]. MeOH additions were carried out by adding MeOH at a concentration of 3 mM (based on Jensen et al. [29]) to a parallel set of samples from Pond C prior to amendments.

### Modeling N removal

A simplistic model was constructed to estimate the mean dry season N removal (NR) capacity (%) of the four settlement ponds. NR was estimated using the potential N_2_ production rates calculated in the present study, and N inputs into the pond through the wastewater. Given the substantial contribution of N remineralized from sludge in shrimp grow-out ponds (often exceeding inputs of N originating from feeds [30]), a variable to account for remineralization inputs was also added (N*_imin_*). The following equation was used to calculate N removal and the parameters are further defined in Table 1:

**Table 1:** An estimate of nitrogen inputs and microbial removal from settlement ponds, note TN = total nitrogen, WW = wastewater, min = mineralization.

Parameter	Value	Unit	Reference
Pond area	6000	m^2^	Farm proprietors Pers. comm.
Mean TN WW input	14.8	kg N d^−1^	EPA monitoring data
Mean net NH_4_^+^ min	27.8	mmol m^−2^ h^−1^	[30]
Mean net DON min	0.6	mmol m^−2^ h^−1^	[30]
Mean N_2_ production	2.9	nmol N cm^−3^ h^−1^	Slurry assay
Net N removal	2.5	%	Model

#### Equation 1



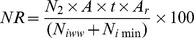
where N_2_ = the mean total (inclusive of anammox) N_2_ production rate measured during the 24 h incubation (nmol N cm^−3^ h^−1^; Table 1). We adopted a conservative approach and assumed that N_2_ production, driven by denitrification, only occurs in the top 1 cm of the sediment. Denitrification occurs at the oxic-anoxic interface so the depth at which it occurs is dependent on O_2_ penetration into the sediments. O_2_ penetration is estimated at <0.5 mm in fish farm wastewater treatment ponds [4], 1.5–4 mm in sediments below fish cages and associated reference sites and up to 20 mm in a muddy macrotidal estuary [31]. This active zone is subsequently extrapolated to estimate rates for the entire area of the settlement pond. The remaining parameters are defined as follows: *A* = mean area of the settlement pond (m^2^); *t* = 24 (h d^−1^); *A_r_* = atomic weight of N; N*_iww_* = mean rate of TN input (inclusive of particulates and dissolved) via the wastewater (environmental protection agency (EPA) monitoring data, quantified monthly by Farm 1; kg N d^−1^); N*_imin_* = mean rate of N input via mineralisation (deduced from NH_4_^+^ and DON fluxes in Burford and Longmore [32]; Table 1; kg N d^−1^).

### Calculations and statistical analysis

The sediment characteristics data was analysed as a 2-factor nested design, pond and zone(pond) using permutational multivariate analysis of variance (PERMANOVA) [33]. PERMANOVA calculated *p*-values from 9999 permutations based on Bray-Curtis distances. A 1-factor PERMANOVA was subsequently used to compare differences in N_2_ production rate data (three variables; denitrification, anammox and total N_2_ production) between ponds with zones as replicates (*n* = 3). 9999 permutations were again used to calculate *p*-values based on Bray-Curtis distance. PRIMER version 6 and PERMANOVA+ version 1.0.4 were used to conduct both analyses.

The relationship between N_2_ production rate (three variables: denitrification, anammox and total N_2_ production) and sediment characteristics was subsequently investigated using the BIOENV procedure in PRIMER. This procedure performs a rank correlation of the two similarity matrices (described above) and tests every combination of sediment characteristics to determine which set of variables best explains the observed N_2_ production rates [34]. A Bray-Curtis similarity matrix comprised of both N_2_ production rate data and sediment variable data was also used to conduct a hierarchical agglomerative cluster analysis which was superimposed on a multidimensional scaling (nMDS) plot. The nMDS plot provided a 2-D visualization of the relationship between sediment characteristics and N_2_ production rates.

The effect of carbon addition on potential N_2_ production rate in sediments was analyzed with paired *t*-Tests for each carbon source (POM and MeOH).

## Results

### Pond characteristics and abiotic factors

Surface water temperature (25.8°C±1.0) and pH (7.6±0.2) varied little similar across all ponds and zones. Surface water salinity in Pond C (Farm 2) was lower (17–18‰) than the other three ponds (31–35‰; Table 2) due to its location in the wet tropics where precipitation is high (Figure 1).

**Table 2:** Mean surface water salinity (*n* = 3±1 SE) and abiotic sediment characteristics (*n* = 9±1 SE) in the four settlement ponds (A, B, C and D) used to treat aquaculture wastewater (µmol g^−1^ unless stated).

	Pond A	Pond B	Pond C	Pond D
Salinity (‰)	31±0	34±0	18±0	35±0
Porosity (%)	0.5±0.0	0.5±0.0	0.5±0.0	0.6±0.0
TOC	61±13	62±6	43±5	63±4
TOC (%)	0.7±0.9	0.8±0.1	0.5±0.1	0.8±0.1
TN	5±1	6±1	4±1	8±1
TN (%)	0.1±1.0	0.1±0.4	0.1±0.8	0.1±0.6
TP	18±4	14±2	5±1	14±3
S	9±1	9±2	12±2	9±0
Fe	43±6	52±5	18±2	25±1
Mn	8±2	6±1	1±0	2±0

Sediment at all zones was uniformly dark black with minor color variation shown in a narrow lighter band (∼3 mm oxic zone) at the surface of the sediment. The porosity ranged between 41–72% (Table 2) and sediments produced a rich hydrogen-sulfide smell and gaseous bubbles (presumably consisting of a mix of biogases) at the water surface when the sediment was disturbed. Very little bioturbation by burrowing organisms or flora was evident. There was significant variability between ponds (Table 3; PERMANOVA; pond; *Pseudo F* = 2.06, *P* = 0.028) and between zones within ponds (Table 3; PERMANOVA; zone (pond); *Pseudo F* = 33.83, *P*<0.001). The variance in sediment characteristics at the finer scale (i.e. meters) between zones within ponds (52.4%) was greater than the variance between settlement ponds located kilometers apart (31.6%).

**Table 3:** A summary of statistical analyses; PERMANOVAs based on the Bray-Curtis similarities of transformed (4^th^ root) sediment characteristic data and potential N_2_ production rate data.

Sediment characteristics				
Test				PERMANOVA
Factors	df	MS	Pseudo-*F*	*P*
Pond	3	39	2.06	0.028
Zone (Pond)	8	19	33.83	0.000

N_2_ production rate				
Test				PERMANOVA
Factors	df	MS	Pseudo-*F*	*P*
Pond	3	4029	3.91	0.001

### Denitrification and anammox potential

**Figure 2:**
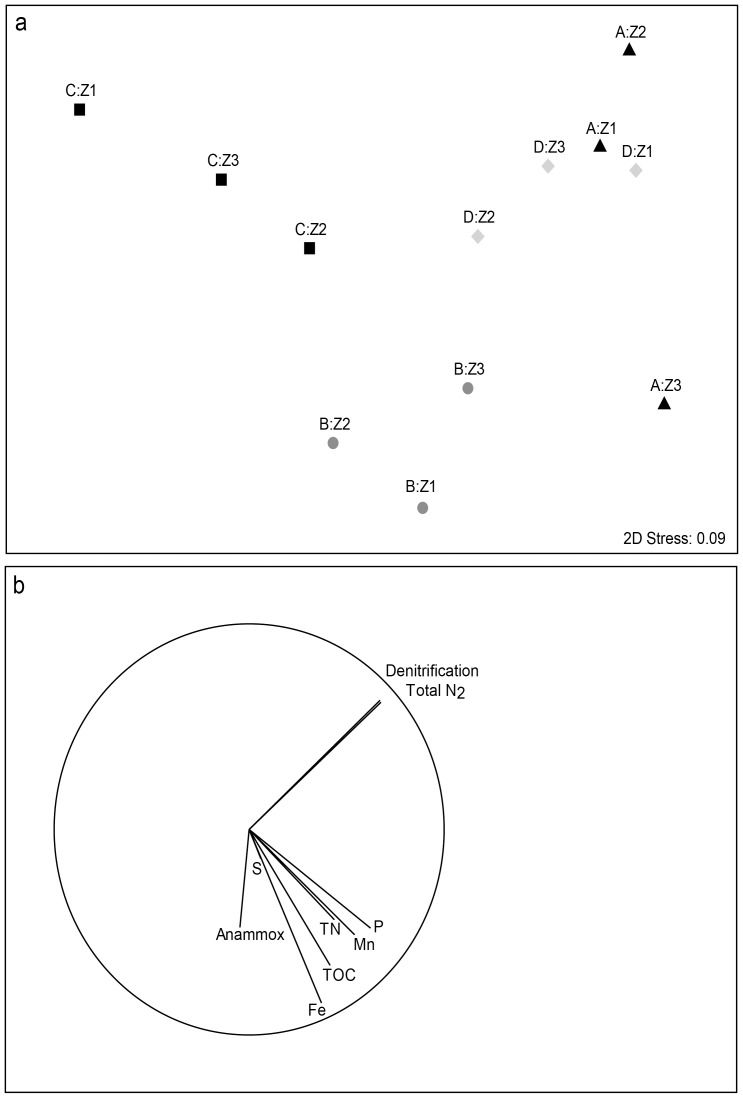
Similarity between N_2_ production rates and sediment characteristics in the four settlement ponds. a) nMDS ordination; 2-D stress = 0.09. b) The same nMDS as a), with vectors superimposed, the length and direction of which indicated the strength of the correlation and direction of change between the two nMDS axes.

There was also a significant difference in the potential rate of N_2_ production between ponds (Table 3; PERMANOVA; pond; *Pseudo F* = 3.91, *P* = 0.001). The potential rate was highest in sediments collected from pond A, with denitrification the sole producer of N_2_ (7.07±2.99 nmol N cm^−3^ h^−1^; Table 4) and lowest in sediments collected from pond C, where again denitrification was the responsible for 100% of the N_2_ produced (0.004±0.003 nmol N cm^−3^ h^−1^; Table 4). However, there was no correlation between the potential production of N_2_ in zones within ponds and different sediment characteristics that defined each pond (nMDS, Figure 2a & b). For example, pond B zone 3 had the highest anammox rates and low denitrification, whereas pond A, zones 2 and 3 had the opposite trend (Figure 2a). This is highlighted in the vector loadings for which the vectors for anammox and denitrification are clearly negatively correlated (Figure 2b).

**Table 4:** The rate (nmol N cm^−3^ h^−1^) of N_2_ production in three incubations (i.e. 24 h, 1.5 h and in the incubation with carbon additions).

Pond	24 h incubation		1.5 h incubation		Carbon Incubation	
	DNT	ANA	DNT	ANA	DNT	ANA
A	7.07±2.99	ND			7.97±3.35	ND
B	0.06±0.06	0.22±0.12				
C	0.004±0.003	ND			0.004±0.003	0.03±0.02
D	4.36±2.01	ND	6.32±4.16	0.48±0.48		

DNT = denitrification; ANA = anammox.

Highly positive or negative loadings of the sediment characteristics appeared to have little influence on total N_2_ production or denitrification (Figure 2b) as these are perpendicular to the positive loadings of all the sediment characteristics. Anammox did cluster near sediment variables (Figure 2b), however there was no correlation between the N_2_ production matrix (inclusive of total N_2_, denitrification and anammox) or the sediment variable matrix (BIOENV analysis; *ρ* = 0.134, *P* = 0.730).

**Figure 3:**
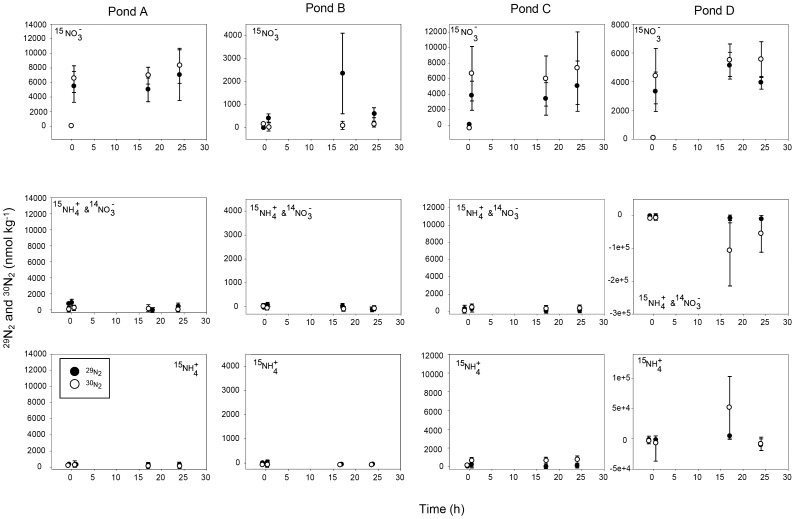
Production of ^29^N_2_ (black circles) and ^30^N_2_ (white circles) over 24 h. ^15^N-N_2_ production in the presence of ^15^N-NO_3_^−^ is represented in row 1, ^15^N-N_2_ production in the presence of ^15^N-NH_4_^+^ and ^14^N-NO_3_^−^ is represented in row 2 and ^15^N-N_2_ production in the presence of ^15^N-NH_4_^+^ is represented in row 3. Column 1 represents ^15^N-N_2_ production in sediments collected from pond A, column 2 represents ^15^N-N_2_ production in sediments collected from pond B, column 3 represents ^15^N-N_2_ production in sediments collected from pond C and column 4 represents ^15^N-N_2_ production sediments collected from pond D.

In incubations with ^15^N labeling of nitrate only, the majority of ^15^N-NO_3_^−^ converted to N_2_ was found in ^30^N_2_ (Figure 3). Only in pond B was more of ^15^N-NO_3_^−^ that was converted to N_2_ found in ^29^N_2_ than in ^30^N_2_ (Figure 3). Anammox was detected in pond B sediments as indicated by the higher percent recovery (0.67±0.28%) of ^15^N-N_2_ in treatments where ^15^N-NH_4_^+^ and unlabelled ^14^N-NO_3_^−^ were added compared to treatments where ^15^N-NH_4_^+^ was added (0.28±0.09%; Table 5). However, in this pond total recovery of ^15^N-NO_3_^−^ as ^15^N-N_2_ was extremely low (0.20±0.07; Table 5).

**Table 5:** The percent recovery of added ^15^N as labelled N_2_ in three treatments.

	^15^N-NO_3_^−^	^15^N-NH_4_^+^ & ^14^N-NO_3_^−^	^15^NH_4_^+^
A	11.8±1.17	0.00±0.00	0.00±0.00
B	0.20±0.07	0.67±0.28	0.28±0.09
C	10.92±1.99	0.26±0.08	0.43±0.07
D	8.79±0.61	0.01±0.32	0.00±0.00

### Slurry assay with carbon manipulation

There was no significant difference in the rate of N_2_ production when either POM (Table 3; Pond A; paired *t*-Test; *P* = 0.350, *n* = 3) or methanol (Table 3; Pond C, paired *t*-Test; *P* = 0.744, *n* = 3) was added to the experimental sediment slurries (Table 4; 24 h incubation compared to carbon incubation).

### Nitrogen removal capacity

We estimate that 2.5% of the total N inputs to the settlement pond are removed through denitrification and anammox (Table 1).

## Discussion

### Total N_2_ production and controlling mechanisms

Isotope tracer techniques confirmed the production of N_2_ in sediment collected at all three zones within each of the four settlement ponds used to treat wastewater from commercial prawn and barramundi farms. The potential rates (0–7.07 nmol N cm^−3^ h^−1^) were within the range of those reported for a subtropical constructed wetland (1.1±0.2 to 13.1±2.6 nmol N cm^−3^ h^−1^) [20], but lower than those reported for subtropical mangrove and shrimp grow out pond sediments (21.5–78.5 nmol N cm^−3^ h^−1^) [35]. Nevertheless, it can be assumed that both denitrifying bacteria and *Planctomycetes* (anammox bacteria) are present in the ponds and that there is potential to stimulate N_2_ production rates and enhance N removal. To achieve this, an understanding of the mechanisms controlling N_2_ production is required. We therefore investigated the effect of carbon additions on N_2_ production rate and the relationship between the concentration of sediment elements and N_2_ production rates. However, there was no significant change in the rate of N_2_ production under carbon loading and there was no correlation between any of the measured sediment variables and N_2_ production rate via denitrification or anammox.

Denitrification is often limited by carbon in aquaculture ponds, as carnivorous marine species require high inputs of protein rich feeds. N removal can be enhanced through the addition of an exogenous carbon source, for example glucose and cassava meal [26] or molasses [25] have been added to shrimp farm wastewater treatment processes, resulting in up to 99% removal of NH_4_^+^, NO_3_^−^ and NO_2_^−^. Similarly, methanol is a common additive to enhance denitrification for municipal wastewater treatment, increasing degradation of NO_2_^−^ in activated sludge from 0.27 mg NO_2_^−^ g^−1^ volatile suspended solids (VSS) h^−1^ to 1.20 mg NO_2_^−^ g^−1^ VSS h^−1^ [27]. However, in the present study N_2_ production was not enhanced through the addition of carbon, suggesting that there are additional controlling mechanisms driving N_2_ production. This concurs with the lack of significant correlation between measured sedimentary TOC and N_2_ production. The lack of stimulation of N_2_ production after the addition of carbon has also been demonstrated in the oxygen minimum zone of the Arabian Sea, where denitrification (and anammox) was only enhanced at one out of 11 depths [36]. Instead, Bulow et al. [36] highlighted a correlation between denitrification and NO_2_^−^ concentration, a factor which likely also plays a role in controlling denitrification in settlement pond systems but was not measured in the present study. NO_3_^−^ concentration also regulates anammox activity in estuarine sediments [37], so future work should aim to correlate extractable NO_3_^−^, NO_2_^−^ and NH_4_^+^ with denitrification and anammox potentials to determine if these are driving process rates in settlement ponds.

It is also possible that the exogenous carbon source is instead stimulating nitrate ammonifiers (DNRA) and therefore competition for NO*_x_* as a substrate [38]. Of the added ^15^NO_3_^−^ only 7.9±2.7% was recovered as ^15^N_2_, so a large portion (i.e. ∼90%) of added ^15^NO_3_^−^ could be rapidly consumed by competing pathways such as DNRA or assimilation. The prevalence of DNRA or assimilation over denitrification will determine the balance between N being removed from the system through gaseous N_2_ production, or conserved within the system [39]–[41]. Furthermore, although dominance of DNRA over denitrification and anammox has been demonstrated in tropical estuaries [42] and under fish cages [43], DNRA has never been quantified in tropical settlement ponds and warrants further investigation.

Another potential controlling factor may be the presence of free sulfides. Sulfur is cycled rapidly in tropical sediments [44], and is the most important anaerobic decomposition pathway in tropical benthic systems, occurring at rates of 0.2–13 mmol S m^−2^ d^−1^ and releasing free sulfides [45], [46]. Free sulfides inhibit nitrification and therefore may be reducing N_2_ production in the present study by reducing the amount of NO_3_^−^ available to denitrifiers [47]. Additionally, DNRA may be stimulated in the presence of sulfur, increasing competition with denitrifiers for NO_3_^−^ [48]. Again, the effect of sulfur on N_2_ production in tropical settlement ponds is largely unknown and further studies are needed to elucidate the potential of this factor on stifling N removal in settlement ponds.

### Denitrification verses anammox

In our study denitrification was the dominant N_2_ production pathway. In coastal, hyper-nutrified sediments, low N_2_ production through anammox has been attributed to the limitation of NO_2_^−^ [49], [50]. Further controlling factors for anammox are NH_4_^+^, total kilojoule nitrogen, TN, TP, salinity, redox state, and an inverse relationship with TOC [51]. Given these controlling factors anammox potential varies seasonally [51] and reported anammox contribution to N_2_ production is highly variable with values of 1–8% [23], ≤3% [15], 10–15% [52], 19–35% [16], up to 65% [17], 2–67% [22] and 4–79% [53].

Anammox was detected in sediment collected in ponds B (24 h incubation), C (carbon incubation) and D (1.5 h incubation), notably, where overall N_2_ production was exceptionally low. For example, during the 24 h incubation with sediment collected in pond B, N_2_ production was lower than in sediment collected from all other ponds, but anammox contributed 95% to N_2_ production. Low carbon oxidation rates and correspondingly low denitrification (and thus competition for substrate) have been proposed as the reason anammox contribution is high in environments where denitrification is low [17]. Bulow et al. [36] demonstrated that high anammox rates corresponded with low denitrification rates at one site in the oxygen minimum zone in the Arabian Sea. At this site both anammox and denitrification were stimulated by the addition of organic carbon. This suggests that N_2_ production was carbon limited giving anammox the competitive advantage. In tropical estuary systems where high temperatures, low sediment organic content and low water column NO_3_^−^ concentrations prevail, the order of NO*_x_* reduction pathways is proposed to be DNRA>denitrification>anammox [42].

The apparent detection of anammox in the presence of MeOH in sediments collected from Pond C is unusual given that anammox is inhibited by MeOH [28]. It is possible that during the 24 h incubation ^15^NH_4_^+^ was transformed through anoxic nitrification [54], producing ^15^NO_3_^−^ and the resulting ^15^N_2_ was produced as the result of denitrification.

### Settlement pond functioning and implications

Microbial N_2_ production has the potential to play a major role in removing N from aquaculture wastewater. However, we estimated that only 2.5% of total N added to the settlement pond via wastewater inputs and mineralization is removed through N_2_ production. It is likely that the noxious compounds of H_2_S and NH_4_^+^ are produced in settlement ponds when they are left unmanaged with no removal of settled particulate organic matter (sludge). These compounds have significant consequences for the inhibition of microbial processes that remove N from wastewater. In addition, H_2_S accumulation causes a shift in the species of gaseous N produced from N_2_ to N_2_O due to the inhibition of the last step of denitrification [41]. This has detrimental consequences for global warming as N_2_O is ∼300 times more potent than CO_2_ as a greenhouse gas whereas N_2_ is relatively inert [55]. Future research should determine the concentration of H_2_S at which the last reductive step of denitrification is inhibited and relate this to the amount of sludge that has built up in the settlement pond. We recommend that sludge be extracted at this point to prevent H_2_S release and to prevent the recycling of soluble N through mineralization, DNRA or assimilation and subsequent senescence, as has been recommended for grow out ponds previously [30]. Innovative technology, such as anaerobic digesters and biogas capture, is required to convert the large volumes of sludge to a saleable product once removed from the pond. The simple management approach of removing sludge could have the added benefit of decreasing the incidence of competition between DNRA and denitrification thereby optimizing the denitrification and anammox processes for N_2_ production. If N_2_ production could be enhanced to the mean rate reported by Erler et al. [20] from a constructed wetland of 965 µmol N m^−2^ d^−1^, then 100% of total daily N inputs would be removed from settlement ponds every day. However, the estimates in the present study are based on a very simplistic understanding of the settlement pond functioning and the model requires better definition of the parameters. For example, accurate rates of NH_4_^+^ and DON production from the sediments are required to estimate N inputs accurately. Additionally, N_2_ production was measured in the dry season in the present study when rates are likely lower than in the wet season. Wet season precipitation lowers the salinity in the ponds to 5‰ in some cases, which favors higher denitrification, lower DNRA and lower NH_4_^+^ fluxes [56]. Denitrification is further stimulated during periods of heavy precipitation due to increased NO_3_^−^ concentrations from land run-off [43]. An increased understanding of the temporal and spatial variability in N_2_ production rates measured using intact core assays, instead of slurry assays, would also allow accurate predictions of N_2_ production rates. Slurry assays only generate potential rates of N_2_ production and we acknowledge that homogenizing sediments disrupts the sediment profile and can result in different nutrient availability than that which occurs *in situ* [57]. Additionally, an understanding of the rates of competing biogeochemical pathways such as DNRA and assimilation would enhance the accuracy of the model by including N retention rates into the model.

## References

[1] Galloway JN, Townsend AR, Erisman JW, Bekunda M, Cai Z, et al (2008) Transformation of the nitrogen cycle: Recent trends, questions, and potential solutions. Science 320 10.1126/science.1136674 18487183

[2] Thomas Y, Courties C, El Helwe Y, Herbland A, Lemonnier H (2010) Spatial and temporal extension of eutrophication associated with shrimp farm wastewater discharges in the New Caledonia lagoon. Marine Pollution Bulletin 61: 387–398. 20667556 10.1016/j.marpolbul.2010.07.005

[3] Jackson CJ, Preston N, Burford MA, Thompson PJ (2003) Managing the development of sustainable shrimp farming in Australia: the role of sedimentation ponds in treatment of farm discharge water. Aquaculture 226: 23–34.

[4] Bartoli M, Nizzoli D, Naldi M, Vezzulli L, Porrello S, et al (2005) Inorganic nitrogen control in wastewater treatment ponds from a fish farm (Orbetello, Italy): Denitrification versus *Ulva* uptake. Marine Pollution Bulletin 50: 1386–1397. 16045942 10.1016/j.marpolbul.2005.06.011

[5] Archer HE, Mara DD (2003) Waste stabilisation pond developments in New Zealand. Water Sci Technol 48: 9–15. 14510188

[6] Porrello S, Ferrari G, Lenzi M, Persia E (2003) Ammonia variations in phytotreatment ponds of land-based fish farm wastewater. Aquaculture 219: 485–494.

[7] Craggs RJ, Sukias JP, Tanner CT, Davies-Colley RJ (2004) Advanced pond system for dairy-farm effluent treatment. New Zealand Journal of Agricultural Research 47: 449–460.

[8] Bolan NS, Laurenson S, Luo J, Sukias J (2009) Integrated treatment of farm effluents in New Zealand's dairy operations. Bioresource Technology 100: 5490–5497. 19342229 10.1016/j.biortech.2009.03.004

[9] Tanner CC, Sukias J (2003) Linking pond and wetland treatment: performance of domestic and farm systems in New Zealand. Water Sci Technol 48: 331–339. 14510228

[10] de Paula Silva PH, McBride S, de Nys R, Paul NA (2008) Integrating filamentous ‘green tide’ algae into tropical pond-based aquaculture. Aquaculture 284: 74–80.

[11] Erler D, Songsangjinda P, Keawtawee T, Chiayakam K (2007) Nitrogen dynamics in the settlement ponds of a small-scale recirculating shrimp farm (*Penaeus monodon*) in rural Thailand. Aquaculture International 15: 55–66.

[12] Jetten MSM, Wagner M, Fuerst JA, van Loosdrecht M, Kuenen JG, et al (2001) Microbiology and application of the anaerobic ammonium oxidation (‘anammox’) process. Current opinion in biotechnology 12: 283–288. 11404106 10.1016/s0958-1669(00)00211-1

[13] Strous M, Fuerst JA, Kramer EHM, Logemann S, Muyzer G, et al (1999) Missing lithotroph identified as new planctomycete. Nature 400 10.1038/22749 10440372

[14] Nicholls JC, Trimmer M (2009) Widespread occurrence of the anammox reaction in estuarine sediments. Aquatic microbial ecology 55: 105–113.

[15] Koop-Jakobsen K, Giblin AE (2009) Anammox in tidal marsh sediments: The role of salinity, nitrogen loading and marsh vegetation. Estuaries and Coasts 32: 238–245.

[16] Dalsgaard T, Canfield DE, Petersen J, Thamdrup B, Acuña-González J (2003) N_2_ production by the anammox reaction in the anoxic water column of Golfo Dulce, Costa Rica. Nature 422: 606–608. 12686998 10.1038/nature01526

[17] Trimmer M, Nicholls JC (2009) Production of nitrogen gas via anammox and denitrification in intact sediment cores along a continental shelf to slope transect in the North Atlantic. Limmol Oceanogr 54: 577–589.

[18] Avimelech Y (1999) Carbon/nitrogen ratio as a control element in aquaculture systems. Aquaculture 176: 227–235.

[19] Loring DH, Rantala RTT (1992) Manual for the geochemical analyses of marine sediment and suspended particulate matter. Earth Sci Rev 32: 235–283.

[20] Erler DV, Eyre BD, Davison L (2008) The contribution of anammox and denitrification to sediment N_2_ production in a surface flow constructed wetland. Environmental Science & Technology 42: 9144–9150. 19174884 10.1021/es801175t

[21] Canfield DE, Thamdrup B, Hansen JW (1993) The anaerobic degradation of organic-matter in Danish coastal sediments - iron reduction, manganese reduction, and sulfate reduction. Geochimica Et Cosmochimica Acta 57: 3867–3883. 11537734 10.1016/0016-7037(93)90340-3

[22] Thamdrup B, Dalsgaard T (2002) Production of N_2_ through anaerobic ammonium oxidation coupled to nitrate reduction in marine sediments. Applied and environmental microbiology 68: 1312–1318. 11872482 10.1128/AEM.68.3.1312-1318.2002 PMC123779

[23] Trimmer M, Nicholls JC, Deflandre B (2003) Anaerobic ammonium oxidation measured in sediments along the Thames Estuary, United Kingdom. Applied and environmental microbiology 69: 6447–6454. 14602599 10.1128/AEM.69.11.6447-6454.2003 PMC262292

[24] Dalsgaard T, Thamdrup B (2002) Factors controlling anaerobic ammonium oxidation with nitrite in marine sediments Applied and Environmental. Microbiology 68: 3802–3808. 10.1128/AEM.68.8.3802-3808.2002 PMC124030 12147475

[25] Roy D, Hassan K, Boopathy R (2010) Effect of carbon to nitrogen (C∶N) ratio on nitrogen removal from shrimp production waste water using sequencing batch reactor. J Ind Microbiol Biotechnol 37: 1105–1110. 20835881 10.1007/s10295-010-0869-4

[26] Avnimelech Y (1999) Carbon/nitrogen ratio as a control element in aquaculture systems. Aquaculture 176: 227–235.

[27] Adav SS, Lee DJ, Lai JY (2010) Enhanced biological denitrification of high concentration of nitrite with supplementary carbon source. Applied Microbiology and Biotechnology 85: 773–778. 19809812 10.1007/s00253-009-2265-4

[28] Güven D, Dapena A, Kartal B, Schmid MC, Mass B, et al (2005) Propionate oxidation by and methanol inhibition of anaerobic ammonium-oxidizing bacteria. Applied and environmental microbiology 71: 1066–1071. 15691967 10.1128/AEM.71.2.1066-1071.2005 PMC546716

[29] Jensen MM, Thamdrup B, Dalsgaard T (2007) Effects of specific inhibitors on anammox and denitrification in marine sediments. Applied and environmental microbiology 73: 3151–3158. 17369344 10.1128/AEM.01898-06 PMC1907100

[30] Burford MA, Lorenzen K (2004) Modelling nitrogen dynamics in intensive shrimp ponds: the role of sediment remineralization. Aquaculture 229: 129–145.

[31] Dong LF, Thornton DCO, Nedwell DB, Underwood GJC (2000) Denitrification in sediments of the River Colne estuary, England. Marine ecology progress series 203: 109–122.

[32] Burford MA, Longmore AR (2001) High ammonium production from sediments in hypereutrophic shrimp ponds. Marine ecology progress series 224: 187–195.

[33] Anderson MJ, Gorley RN, Clarke KR (2008) PERMANOVA+ for PRIMER: Guide to software and statistical methods. Plymouth: PRIMER-E Ltd.

[34] Clarke KR, Warwick RM (2005) Primer-6 computer program. Natural Environment Research Council: Plymouth.

[35] Amano T, Yoshinaga I, Yamagishi T, Chu VT, Pham TT, et al (2011) Contribution of Anammox Bacteria to Benthic Nitrogen Cycling in a Mangrove Forest and Shrimp Ponds, Haiphong, Vietnam. Microbes Environ 26: 1–6. 21487196 10.1264/jsme2.me10150

[36] Bulow SE, Rich JJ, Naik HS, Pratihary AK, Ward BB (2010) Denitrification exceeds anammox as a nitrogen loss pathway in the Arabian Sea oxygen minimum zone. Deep-Sea Research I 57: 384–393.

[37] Trimmer M, Nicholls JC, Morley N, Davies CA, Aldridge J (2005) Biphasic behavior of anammox regulated by nitrite and nitrate in an estuarine sediment. Applied and Environmental Microbiology 71: 1923–1930. 15812021 10.1128/AEM.71.4.1923-1930.2005 PMC1082541

[38] Yin SX, Chen D, Chen LM, Edis R (2002) Dissimilatory nitrate reduction to ammonium and responsible microorganisms in two Chinese and Australian paddy soils. Soil Biol Biochem 34: 1131–1137.

[39] Burgin AJ, Hamilton SK (2007) Have we overemphasized the role of denitrification in aquatic ecosystems? A review of nitrate removal pathways. Front Ecol Environ 5: 89–96.

[40] King D, Nedwell DB (1984) Changes in the nitrate-reducing community of an anaerobic saltmarsh sediment in response to seasonal selection by temperature. J Gen Microbiol 130: 2935–2941.

[41] Brunet RC, Garcia-Gil LJ (1996) Sulfide-induced dissimilatory nitrate reduction to ammonia in anaerobic freshwater sediments. Fems Microbiology Ecology 21: 131–138.

[42] Dong LF, Sobey MN, Smith CJ, Rusmana I, Phillips W, et al (2011) Dissimilatory reduction of nitrate to ammonium, not denitrification or anammox, dominates benthic nitrate reduction in tropical estuaries. Limmol Oceanogr 56: 279–291.

[43] Christensen PB, Rysgaard S, Sloth NP, Dalsgaard T, Schwaerter S (2000) Sediment mineralization, nutrient fluxes, denitrification and dissimilatory nitrate reduction to ammonium in an estuarine fjord with sea cage trout farms. Aquatic microbial ecology 21: 73–84.

[44] Madrid VM, Aller RC, Aller JY, Chistoserdov AY (2006) Evidence ofthe activity of dissimilatory sulfate-reducing prokaryotes in nonsul¢dogenic tropical mobile muds. FEMS Microbiology Ecology 57: 169–181. 16867136 10.1111/j.1574-6941.2006.00123.x

[45] Alongi DM, Tirendi F, Trott LA, Xuan TT (2000) Benthic decomposition rates and pathways in plantations of the mangrove Rhizophora apiculata in the Mekong delta, Vietnam. . Mar Ecol-Prog Ser 194: 87–101.

[46] Meyer-Reil L-A, Köster M (2000) Eutrophication of marine waters: effects on benthic microbial communities. Marine Pollution Bulletin 41: 255–263.

[47] Joye SB, Hollibaugh JT (1995) Influence of sulphide inhibition of nitrification on nitrogen regeneration in sediments. Science 270: 623–625.

[48] Jørgensen BB (2010) Big sulfur bacteria. ISME Journal 4: 1083–1084. 20631811 10.1038/ismej.2010.106

[49] Dang H, Chen R, Wang L, Guo L, Chen P, et al (2010) Environmental factors shape sediment anammox bacterial communities in hypernutrified Jiaozhou Bay, China. Applied and environmental microbiology 76: 7036–7047. 20833786 10.1128/AEM.01264-10 PMC2976235

[50] Risgaard-Petersen N, Meyer RL, Revsbech NP (2005) Denitrification and anaerobic ammonium oxidation in sediments: effects of microphytobenthos and NO3. Aquatic Microbial Ecology 40: 67–76.

[51] Li M, Cao HL, Hong YG, Gu JD (2011) Seasonal Dynamics of Anammox Bacteria in Estuarial Sediment of the Mai Po Nature Reserve Revealed by Analyzing the 16S rRNA and Hydrazine Oxidoreductase (hzo) Genes. Microbes Environ 26: 15–22. 21487198 10.1264/jsme2.me10131

[52] Hietanen S, Kuparinen J (2008) Seasonal and short-term variation in denitrification and anammox at a coastal station on the Gulf of Finland, Baltic Sea. Hydrobiologia 596: 67–77.

[53] Engstrom P, Dalsgaard T, Hulth S, Aller RC (2005) Anaerobic ammonium oxidation by nitirte (anammox): Implications for N2 production in coastal marine sediments. Geochimica et Cosmochimica Acta 69: 2057–2065.

[54] Hulth S, Aller RC, Gilbert F (1999) Coupled anoxic nitrification manganese reduction in marine sediments. Geochimica Et Cosmochimica Acta 63: 49–66.

[55] IPCC (2001) Climate change 2001: The scientific basis. Cambridge University Press: Cambridge, UK.

[56] Giblin AE, Weston NB, Banta GT, Tucker J, Hopkinson CS (2010) The Effects of Salinity on Nitrogen Losses from an Oligohaline Estuarine Sediment. Estuaries and Coasts 33: 1054–1068.

[57] Minjeaud L, Michotey VD, Garcia N, Bonin PC (2009) Seasonal variation in di-nitrogen fluxes and associated processes (denitrification, anammox and nitrogen fixation) in sediment subject to shellfish farming influences. Aquatic Sciences 71: 425–435.

